# Effects of Hemodialysis on Intraocular Pressure and Ocular Perfusion Pressure

**DOI:** 10.7759/cureus.59138

**Published:** 2024-04-27

**Authors:** Ibrahima L Sarr, Binta Sakho, El Hadji Coumba Diouf Guisse, Rokhaya Fall

**Affiliations:** 1 Nephrology, Ouakam Military Hospital, Dakar, SEN; 2 Nephrology, Heinrich Lübke Regional Hospital, Diourbel, SEN; 3 Ophthalmology, Heinrich Lübke Regional Hospital, Diourbel, SEN

**Keywords:** glaucoma, hemodialysis, effects, ocular perfusion pressure, intraocular pressure

## Abstract

Introduction: Elevated intraocular pressure (IOP) and reduced ocular perfusion pressure (OPP) related to hemodialysis (HD) are risk factors for the development and progression of glaucoma. The aim of this study was to evaluate the effect of HD on IOP and OPP in our patients.

Methods: This was a cross-sectional, descriptive, and analytical study conducted between January 2 and February 5, 2024, in the HD and ophthalmology departments of the Heinrich Lübke Regional Hospital in Diourbel. The IOP of both eyes was measured one hour before the start of the HD session and within 30 minutes after the end of the session, by the same ophthalmologist, for all included patients, using a Goldmann applanation tonometer. OPP were calculated and other socio-demographic data collected.

Results: Fifty-eight eyes from 29 chronic HD patients were included. The mean age of the patients was 47.58±12.94 years, with a predominance of women (17 women or 58.6%; sex ratio 0.71). In the left eye, mean IOP increased significantly from 12.21±2.96 mmHg before the HD session to 14.10±4.27 mmHg at the end of the session (p=0.04). For the right eye, the IOP also increased with a strong tendency to significance from 12.97±3.79 mmHg before the HD session to 15.03±5.23 mmHg at the end of the session (p=0.05). OPP did not change significantly after the HD session.

Conclusion: The HD session significantly increased IOP with no significant change in OPP.

## Introduction

Changes in intraocular pressure (IOP) during hemodialysis (HD) are one of the ophthalmological abnormalities that can be observed in chronic HD patients [1]. The effects of HD on IOP have been studied for almost 60 years. Sitprija and Holmes were the first to report an increase in IOP during HD in 1962 [2]. Since then, several studies assessing the effect of HD on IOP have shown different findings and drawn various conclusions. Some studies reported an increase in IOP during HD [2,3], while others reported a significant decrease in IOP during HD [4-6]. However, more recent studies didn't find any significant differences in IOP measurements after HD. They therefore suggested that improvements in dialysis techniques, such as high-flux HD or hemofiltration and more effective urea control, maintain optimal osmolar balance and prevent marked IOP elevation [7-9], which is an important factor in the development and progression of glaucoma. For this reason, specific precautions are always essential for patients with ocular pathologies (e.g., glaucoma) whose vision can be impaired by significant fluctuations in IOP.

Besides, sufficient oxygenation of ocular tissues also requires ocular perfusion pressure (OPP) to be maintained by the systemic regulation of blood pressure (BP), which varies during the HD session. An abrupt change in OPP can lead to optic nerve ischemia, the potential cause of developing glaucoma.

Nevertheless, these issues remain of little interest to ophthalmologists and nephrologists, particularly in our region, where no studies have been published in this area. The aim of the present study was to evaluate the effect of HD on IOP and OPP in our patients.

## Materials and methods

This was a cross-sectional, descriptive, and analytical study carried out between January 2 and February 5, 2024, in the HD and ophthalmology departments of the Heinrich Lübke Regional Hospital in Diourbel. All patients over 18 years of age who had been receiving regular HD for more than three months and who consented to participate in the study were included. In contrast, patients with keratitis, conjunctivitis, blepharitis, corneal scarring, or bulbar phthisis at the time of the study were not included.

After giving their consent, the patients enrolled had IOP measured in both eyes one hour before the start of the HD session and 30 minutes after the end of the session. These measurements were performed by the same ophthalmologist for all patients using a Goldmann applanation tonometer, which is the gold standard for IOP assessment. They all underwent HD for four hours with the same type of Bbraun generator. The composition and flow rate of the dialysate were the same for all patients during the index HD session. The other parameters of the session were defined according to the patient's clinical condition. At the end of the HD session, symptoms that could indicate a change in IOP such as visual blur, eye pain, and headache were collected.

We used the following formula to calculate mean arterial pressure (MAP) before and after the HD session: MAP=diastolic BP+1/3(systolic BP-diastolic BP). Systolic and diastolic BP were measured using an electronic BP monitor placed on the arm without the arteriovenous fistula, with the patient lying in supine position. The OPP was calculated as follows: OPP=MAP-IOP. Systolic ocular perfusion pressure (SOPP), diastolic ocular perfusion pressure (DOPP), and mean ocular perfusion pressure (MOPP) were also calculated using the following formulas: SOPP=SBP-IOP, DOPP=DBP-IOP, and MOPP=2/3(MAP-IOP).

Data analysis was performed using IBM SPSS Statistics for Windows, Version 28.0 (Released 2021; IBM Corp., Armonk, New York, United States). Quantitative data were presented as means and standard deviations and qualitative data as counts and relative frequency. Comparisons between mean IOP, MAP, and OPP before and after the HD session were performed using two-tailed t-tests. The Pearson correlation coefficient was calculated and used to establish the linear correlations. All test results were assessed at the 5% significance level (p≤0.05).

## Results

The study included 58 eyes from 29 chronic HD patients. The mean age of the patients was 47.58±12.94 years, with a predominance of women (17 women or 58.6%; sex ratio 0.71). Hypertensive nephropathy (16 patients, 55.2%) was the most common cause of chronic kidney disease. The mean duration of HD was 35.0±28.7 months. None of the patients had a previous diagnosis of glaucoma. During the HD session, eight patients (27.6%) complained of headache, three patients (10.3 %) reported visual blur, and one patient had ocular pain. Other patient characteristics are shown in Table 1.

**Table 1:** Clinical characteristics of the 29 participants ADPKD: autosomal dominant polycystic kidney disease

Characteristics	n (%)	Mean±standard deviation
Age (years)		47.58±12.94
Women	17 (58.6)	
Hypertensive nephropathy	16 (55.2)	
Nephropathy of undetermined cause	8 (27.6)	
Diabetic nephropathy	1 (3.4)	
ADPKD	1 (3.4)	
Other	3 (10.3)	
Duration of hemodialysis (months)		35.0±28.7
Ultrafiltration (ml/h)		612±178.16
Headache	8 (27.6)	
Vision blur	3 (10.3)	
Eye pain	1 (3.4)	

For the left eye, mean IOP increased significantly from 12.21±2.96 mmHg before the HD session to 14.10±4.27 mmHg at the end of the session (p=0.04). For the right eye, the IOP also increased with a strong tendency to significance from 12.97±3.79 mmHg before the HD session to 15.03±5.23 mmHg at the end of the session (p=0.05) (see Table 2). There was no significant correlation between changes in IOP and the hourly ultrafiltration rate (r=0.06; p=0.75 for the right eye and r=0.15; p=0.41 for the left eye).

**Table 2:** Pressures measured and calculated in the 29 participants before and after the hemodialysis session IOP: intraocular pressure; MAP: mean arterial pressure; OPP: ocular perfusion pressure; SOPP: systolic ocular perfusion pressure; DOPP: diastolic ocular perfusion pressure; MOPP: mean ocular perfusion pressure

Pressure (mmHg)	Before hemodialysis	After hemodialysis	p
MAP	123.62	121.93	0.61
IOP (right eye)	12.97	15.03	0.05
IOP (left eye)	12.21	14.10	0.04
OPP (right eye)	110.66	106.90	0.26
OPP (left eye)	111.41	108.17	0.38
SOPP (right eye)	152.62	148.52	0.29
SOPP (left eye)	153.52	149.79	0.35
DOPP (right eye)	90.10	87.07	0.37
DOPP (left eye)	90.86	87.66	0.36
MOPP (right eye)	73.41	70.93	0.27
MOPP (left eye)	73.86	71.76	0.39

Regarding the different OPPs, we didn't note any difference before and after HD (see Table 2).

## Discussion

The effect of HD on OPP has not been well explored. On the other hand, the effect of HD on IOP has been widely studied, but the findings are not conclusive. These contradictions may be explained by the lack of uniformity in terms of the techniques used to measure IOP, the time of measurement, the condition of the patients, and the presence or absence of glaucoma.

In our study, we showed a statistically significant increase in IOP at the end of the HD session with no significant variation in the different OPPs. Early studies [2,10,11] investigating the effect of HD on IOP mainly reported an increase in IOP, which was explained as the result of a rapid decrease in plasma osmolarity and/or a relative increase in intracellular versus extracellular urea concentration. This rapid shift causes a gradient between plasma and the ocular compartments, leading to a transfer of extracellular fluid from the blood to the anterior chamber. However, more recent studies [12-14] haven't demonstrated any significant change in IOP with HD or a decrease in IOP. They thus suggest that improved urea control with enhanced dialysis techniques prevents a marked rise in IOP [7-9].

In addition, the removal of fluid during ultrafiltration without the concomitant removal of albumin increases colloid osmotic pressure, resulting in a shift of fluid from the aqueous humor to the plasma and a decrease in IOP [12]. In our study, no correlation was found between ultrafiltration and changes in IOP.

Despite numerous reports with diverse findings ranging from an increase to a decrease in IOP during HD, IOP tends to increase in glaucoma patients [15,16]. However, in our study, no patient was diagnosed with glaucoma.

Regarding the effect of HD on OPP, our results concur with those of Barbosa et al. [13]. On the other hand, Hu et al. [17] reported a significant decrease in all the OPP after the HD session compared with the OPP calculated before the session. These differences may be explained from a methodological point of view, as the techniques used to measure IOP and BP are different from those used in our study.

Our study is unique in that it is one of the first in Africa to focus on changes in IOP and OPP after HD sessions. However, it has a number of limitations. Firstly, the small size of our sample is explained by the fact that this was a single-center study. In addition, our patients didn't have any previous ophthalmological follow-up for the diagnosis of glaucoma. Finally, pressures were only measured during one HD session. Repeated measurements over several HD sessions would allow us to see whether the results are reproducible in the same patients.

## Conclusions

In this study, we have shown that IOP increases after the HD session without any change in OPP. We suggest more collaboration between nephrologists and ophthalmologists in order to better manage especially glaucoma patients who may be adversely affected by these changes.
